# A multi-source fusion and feedback-optimized intelligent agent for crop disease and pest diagnosis and treatment

**DOI:** 10.3389/fpls.2026.1811772

**Published:** 2026-06-19

**Authors:** Yimin Xia, Jia Lv, Yuqing He, Fuzhong Li

**Affiliations:** School of Artificial Intelligence, Faculty of Software Technologies, Shanxi Agricultural University, Jinzhong, Shanxi, China

**Keywords:** farmer feedback, intelligent diagnosis and treatment, knowledge graph, multi-source knowledge fusion, reinforcement learning

## Abstract

**Introduction:**

Crop diseases and pests pose a critical threat to global food security and agricultural sustainability. Traditional control methods are often limited by delayed diagnosis and a lack of capability for personalized solutions.

**Methods:**

To address these challenges, we developed a knowledge-enhanced diagnostic and treatment agent, optimized through multi-source knowledge fusion and farmer feedback. The agent integrates disease identification results from a visual model, multi-factor contextual parameters, and a crop knowledge graph. These components form a unified multi-source knowledge representation. The system converts multi-criteria farmer evaluations into reward signals, enabling continuous optimization of action strategies through interaction with real-world environments. Under the combined guidance of multi-source knowledge and farmer feedback-driven reinforcement learning, the agent can generate accurate and practically applicable treatment recommendations without additional task-specific fine-tuning of the generative model.

**Results:**

Experiments on multiple baseline models demonstrate that combining these components consistently achieves the best performance. BERTScore increases by 25.23% on average, accuracy based on large language model evaluation improves by 30.27%, and practicality increases by 37.67%.

**Discussion:**

These results validate the effectiveness and generalization capability of the proposed method.

## 1 Introduction

Staple crops supply the majority of global dietary calories and form the backbone of agricultural production systems Saccone and Vallino (2025). However, these crops face increasing threats from pests and diseases, which pose significant risks to yield stability and food security Godfray et al. (2010). Pests and diseases cause estimated annual economic losses exceeding US$220 billion Savary et al. (2019). This challenge is further exacerbated by climate variability. As global temperatures rise, the survival rates and spatial distributions of harmful organisms are shifting. This intensifies pressure on agricultural systems. Projections indicate that for every 1°C increase in global surface temperature, yield losses for staple crops could rise by 10% to 25% Zhao et al. (2017). Such yield volatility destabilises food supply chains and leads to global price 29 fluctuations Ray et al. (2013); Taghizadeh-Hesary et al. (2019).

In current management practices, chemical intervention remains a potent method for yield preservation. However, its efficacy is often compromised by a lack of precise diagnostic and decision-making guidance. This deficiency frequently results in inefficient pesticide application and increased production costs. Furthermore, indiscriminate agrochemical use can accelerate pathogen resistance and negatively impact soil health and the regulatory functions of agroecosystems Carvalho (2017); Tang et al. (2021). Delays in traditional manual monitoring further worsen the problem, as sudden, large-scale outbreaks are often not captured in time to support precise intervention Liakos et al. (2018). Together, these limitations highlight the challenges of conventional control methodologies in highly dynamic and stochastic field environments.

Extensive research has shown that data-driven precision decision-making systems can optimize the allocation of control resources. These systems enhance pesticide utilisation efficiency while safeguarding crop yields Kamilaris and Prenafeta-Boldú (2018); Klerkx et al. (2019); Iaksch et al. (2021). Moreover, integrating artificial intelligence into pest and disease management provides a promising pathway to balance agricultural productivity with environmental sustainability Aijaz et al. (2025). Although existing data-driven models perform well in controlled laboratory settings, their diagnostic accuracy often degrades under complex real-world field conditions. This highlights the urgent need for robust multi-source data integration Araujo et al. (2023). Empirical evidence suggests that deep fusion of ground-based sensing and meteorological data can substantially reduce misdiagnosis caused by information scarcity or noise Liu and Wang (2024). Crucially, incorporating farmer feedback to optimize action strategies has been shown to significantly improve the treatment reliability of recommendations in real-world agricultural environments Breure et al. (2024); Gautron et al. (2024). Consequently, developing a self-adaptive diagnostic agent not only provides a reliable tool for disease and pest control but also facilitates the transition of smart agriculture from passive monitoring to active decision-making Eastwood et al. (2019).

**Figure 1:**
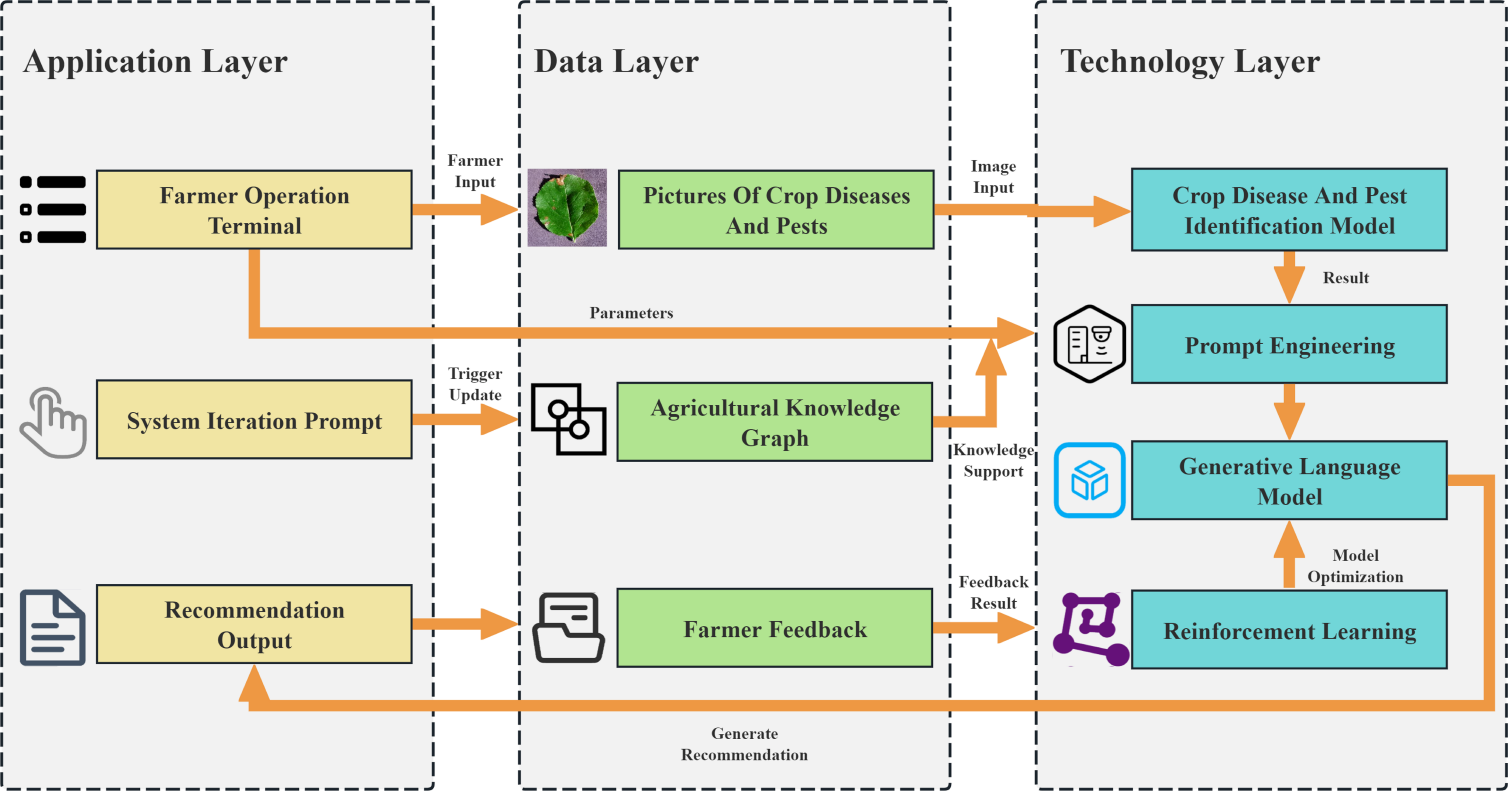
Overall workflow diagram of the crop disease and pest diagnosis and treatment agent.

To address these challenges, this study develops a knowledge-enhanced diagnostic and treatment agent, optimized through multi-source knowledge (MK) and farmer feedback-driven reinforcement learning (RL), as illustrated in **Figure 1**. The agent integrates disease identification results from a visual model, multi-factor contextual parameters, and a knowledge graph, which together constitute the multi-source knowledge. By converting farmer feedback into reinforcement learning reward signals, the agent can iteratively optimize its action strategies. The agent then combines the multi-source knowledge and action strategies into structured inputs to generate personalized treatment recommendations, thereby enhancing the treatment reliability and practical applicability of the generative model in real-world agricultural scenarios. The specific contributions of this paper are as follows:

1. We constructed a structured knowledge graph (KG) to support crop disease and pest management. The KG comprises 7 entity types, 11 attribute types, and 7 relationship types, serving as the core of the multi-source knowledge and providing reliable domain knowledge for the generative model.
2. We developed a multi-source knowledge fusion framework that integrates disease identification results from the visual model, multi-factor contextual parameters, and the knowledge graph into structured inputs. Experiments on three baseline models show that introducing the framework alone improves performance: BERTScore increases by an average of 9.65%, accuracy based on large language model evaluation improves by 19.67%, and practicality increases by 17.8%.
3. We incorporated farmer feedback-driven reinforcement learning to iteratively optimize action strategies. When combined with the multi-source knowledge framework, the generative model achieves the highest performance in treatment accuracy and practicality. Experiments show further improvements: BERTScore increases by 25.23%, accuracy based on large language model evaluation improves by 30.27%, and practicality increases by 37.67%, demonstrating the synergistic effect of the two components.

## 2 Materials and methods

**Figure 2:**
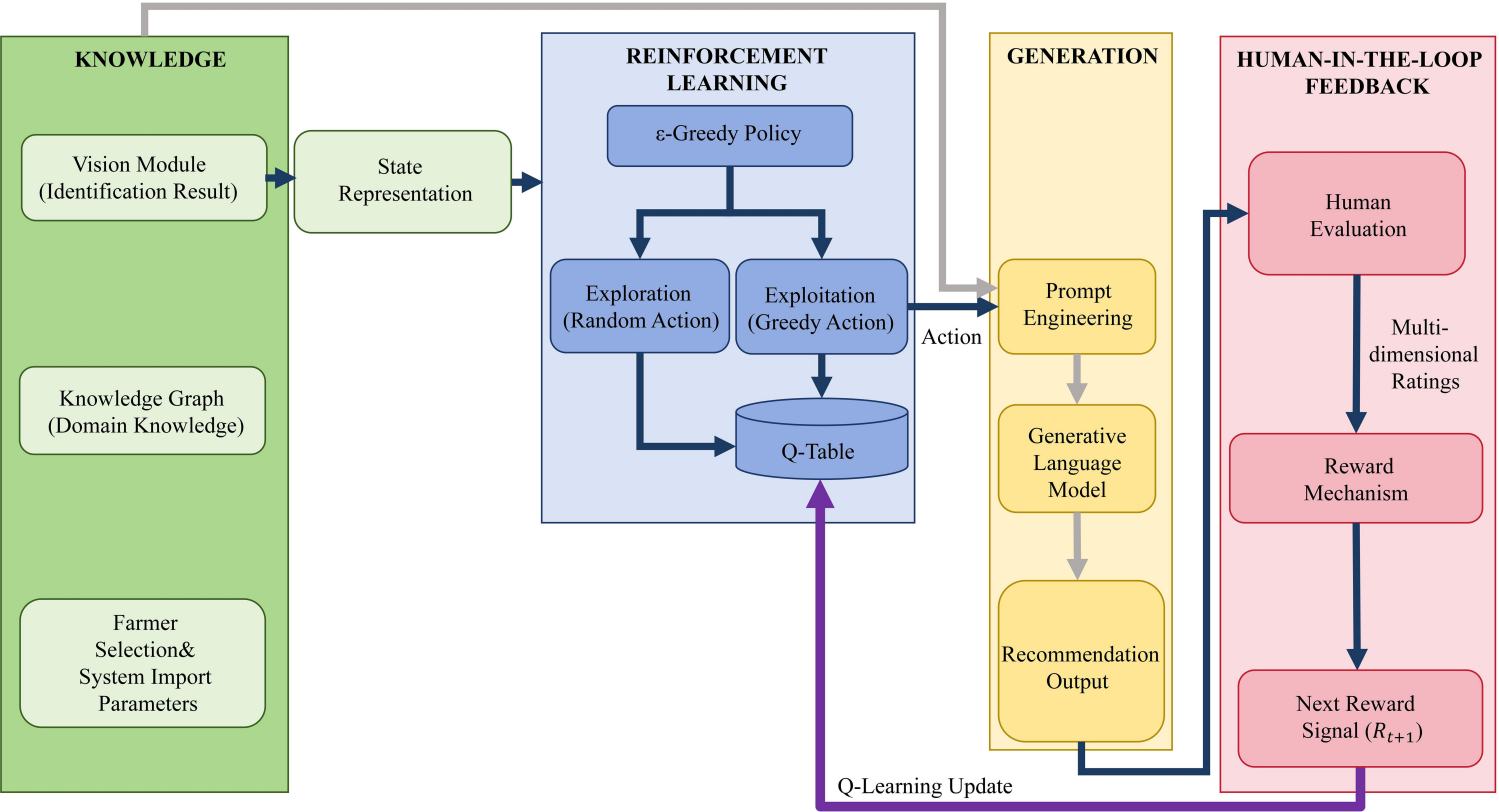
Knowledge-augmented diagnosis and treatment agent optimized by farmer feedback.

This study develops an intelligent agent for diagnostics driven by farmer feedback, using multi-source knowledge fusion. The agent selects an optimal action strategy based on the current disease and integrates multi-source knowledge to generate personalized recommendations. It then updates the action strategy according to multi-criteria farmer feedback, achieving a closed-loop process from knowledge and action decisions to recommendation generation and optimization, as shown in **Figure 2**. Reinforcement learning does not introduce new domain knowledge but optimizes the selection of action strategies based on farmer feedback.

### 2.1 Construction of multi-source knowledge

**Figure 3:**
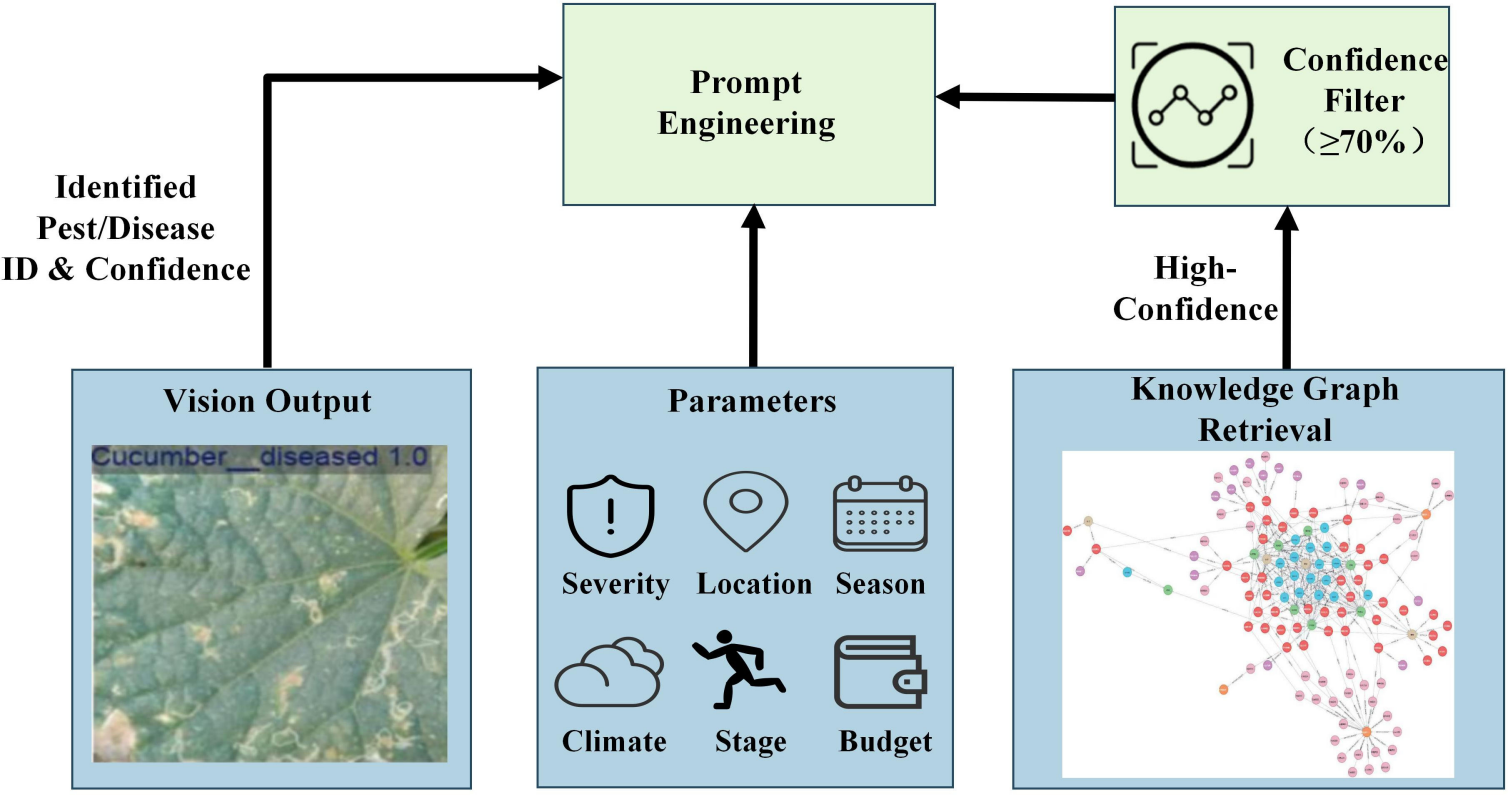
Multi-source knowledge fusion.

As illustrated in **Figure 3**, the generative language model constructs prompts by integrating multiple sources of knowledge, including disease or pest identification results from the vision model, multi-factor contextual parameters, and treatment plans retrieved from the knowledge graph.

Disease or pest results are identified through a vision model. The visual training dataset was compiled from multiple publicly available plant disease image repositories, including PlantVillage Dobrovsky (2021) and distributed via Kaggle Mohanty et al. (2016). It covers crop disease types of significant agricultural value, ensuring both class diversity and practical relevance. The research team integrated annotated resources from 14 public image collections and applied rigorous filtering, resulting in a dataset comprising 88 disease states with over 76,000 images. Although some scene bias remains in certain categories due to varying capture conditions, this dataset provides a robust and generalizable foundation for vision-based disease identification.

The agent presets six core parameters in the farmer interaction interface. Severity and budget are manually selected by the farmer Albahli (2025), while location, season, climate, and crop growth stage are automatically populated. Severity is categorized as mild (<5%), moderate (5–15%), severe (15–30%), and critical (>30%), and budget as low (<1000 USD/acre), medium (1000–2000 USD/acre), and high (>2000 USD/acre).

**Figure 4:**
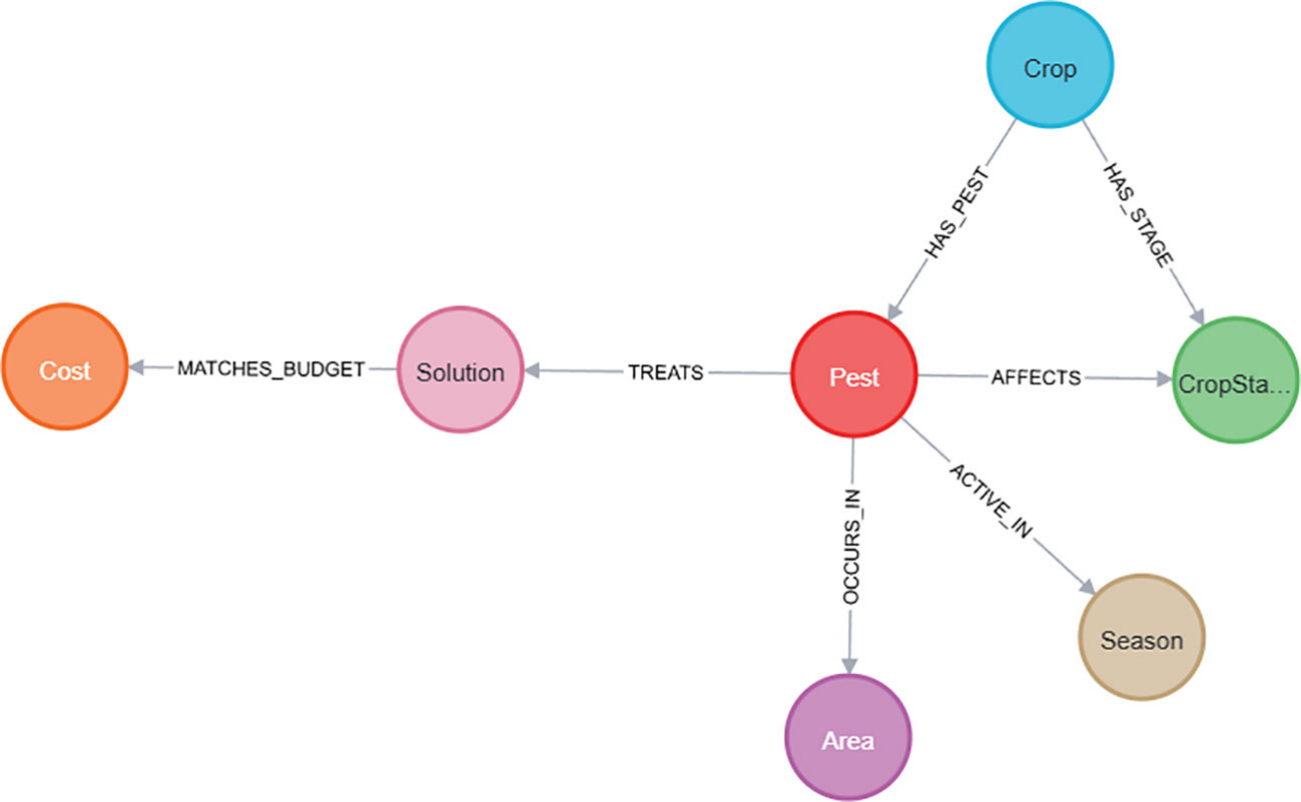
Relationship types.

This study constructs a knowledge graph covering pests or diseases, region, season, crop, cost, control measures, and crop growth stage Zhao et al. (2024). The knowledge graph construction begins with domain ontology design, defining entity, relation, and attribute types according to application requirements. The knowledge graph comprises seven entity types and eleven attribute types, as shown in **Table 1**, and seven relation types, as illustrated in **Figure 4**. The defined relations, as shown in **Table 2**, include crop–pest associations, pest–growth stage interactions, spatial distribution, seasonal activity, treatment mapping, and cost alignment, forming a structured decision-support framework. As shown in **Supplementary Table S1**, specific knowledge is extracted from authoritative and diverse data sources. Entities, attributes, and relations are extracted using a combination of natural language processing and manual annotation. Identical entities across sources are aligned to remove redundancy and inconsistency. Validated triples are stored in a graph database, forming a structured knowledge network that supports subsequent querying, reasoning, and decision-making applications. The outputs from the vision model and these parameters—disease or pest type, region, season, crop, cost, and crop growth stage—serve as the initial input for knowledge graph reasoning. This provides a structured and reliable foundation for generating personalized treatment recommendations.

**Table 1:** Entity types and their attribute types.

Entity type	Attribute type
Pests and Diseases	name, type, id, severity_level, damage_desc
Region	name, type, id, climate_feature
Season	name, type, id, time_range
Crop	name, type, id
Cost	name, id, range, strategy
Crop Growth Stages	name, type, id, key feature
Control Plans	name, type, id, operation_steps

**Table 2:** Relationship types and descriptions.

Relationship type	Relationship description
Crop → HAS_PEST → Pest	Defines potential pests and diseases affecting the crop.
Pest → AFFECTS → CropStage	Defines the specific growth stage at which the pest causes harm.
Pest → OCCURS_IN → Area	Defines the geographical areas where the pest is prevalent.
Pest → ACTIVE_IN → Season	Defines the active season for the pest.
Crop → HAS_STAGE → CropStage	Defines the growth cycle stage of the crop.
Pest → TREATS → Solution	Defines the treatment solution for a specific pest or disease.
Solution → MATCHES_BUDGET → Cost	Defines the cost tier corresponding to the control solution.

The agent integrates disease identification results, multi-factor parameters, and treatment-related knowledge retrieved from the knowledge graph with confidence ≥ 70% into a structured prompt for the generative model. This threshold is used to filter triples that are supported by at least two authoritative sources (Zhang et al., 2025; Ban et al., 2022). This setting aims to balance noise reduction and knowledge coverage, a trade-off widely recognized in knowledge graph construction and quality control (Amaral et al., 2024; Zhang et al., 2024). Without task-specific tuning, this approach effectively filters noisy or ambiguous triples while preserving sufficient valid relations to maintain the integrity of the knowledge network Peng et al. (2026).

### 2.2 State and action space design

The selection of control strategies is modeled as a Markov decision process Fayaz et al. (2022). The agent obtains the output of a visual disease identification model. For each input image, the model produces a single predicted disease label $\hat{d}\in \left\{d_{1},d_{2},\ldots ,d_{88}\right\}$. This predicted label is directly used as the state $s_{t}=\hat{d}$. The confidence score is excluded from the state to reduce state redundancy and improve learning stability, and is instead used as auxiliary information in the prompt construction stage. Thus, the state space S consists of 88 discrete states, each corresponding to a specific pest or disease category. The visual model’s top-1 prediction serves as the sole mapping rule.

Each state is associated with four candidate actions. The action space $A=\left\{a_{1},a_{2},a_{3},a_{4}\right\}$ corresponds to physical control, chemical control, ecological management, and simplified operations, respectively.

For each state *s_t_*, a valid action subset $A_{\text{valid}}\left(s_{t}\right)\subseteq A$ is predefined based on domain knowledge. Three plant protection experts predefined pruning rules based on contraindications for controlling common diseases. Specifically, for the current state *s_t_*, if an action strategy is known to be ineffective or harmful for the given disease, that action is temporarily removed from the $A_{\text{valid}}\left(s_{t}\right)$. For example, chemical control is often ineffective against viral diseases and is therefore pruned.

In each interaction, the agent observes the current state *s_t_*, selects an action $a_{t}\in A_{\text{valid}}\left(s_{t}\right)$, integrates knowledge from multiple sources to generate control recommendations, receives feedback and rewards *R_t_*_+ 1_ from farmers, and then updates the *Q*-value as described in Section 2.4.

### 2.3 Q-table initialization

To initialize the *Q*-table, this study employs a warm-start filling technique. The base scores for the four control strategies are pre-injected by plant protection experts based on general experience in disease control, with initial settings of: *Q*(*s,a*_1_) = 3.0, *Q*(*s,a*_2_) = 4.0, *Q*(*s,a*_3_) = 3.5, and *Q*(*s,a*_4_) = 4.5, ranging from 1 to 5 points. All state-action pairs share the same set of initial scores. This strategy significantly mitigates the randomness of reinforcement learning during the cold-start phase. The above initial values were set based on the following considerations: chemical control and simplified operations are widely used in agricultural production, and farmers generally recognize their effectiveness and feasibility, so they were assigned higher initial scores; although ecological management is environmentally friendly, it takes relatively longer to show results and requires higher operational standards, so its initial score is slightly lower than the first two; physical control is limited by implementation conditions and scope of application and is typically used as a supplementary measure, hence it has the lowest initial score. This setting was determined by averaging independent scores from three plant protection experts, reflecting a general preference for conventional disease control strategies while leaving room for subsequent optimization based on farmer feedbackYuan et al. (2024).

### 2.4 Reward design and Q-learning update

The agent employs the *ϵ*-greedy strategy to balance exploration and exploitation. The exploration rate *ϵ* is set to 0.2, meaning the agent selects a random action from the valid action subset with a 20% probability and selects the currently estimated optimal action with an 80% probability, as shown in Equation 1:

(1) $$a_{t}=\{\begin{array}{ll} \arg\;\max_{a\in A_{\text{valid}}\left(s_{t}\right)}Q\left(s_{t},a\right), & \text{with probability }1-\epsilon \\ \text{randomly sample }a\in A_{\text{valid}}\left(s_{t}\right), & \text{with probability }\epsilon \end{array}$$

The selected action strategy is embedded as a style constraint into the prompt template. The template uses the phrase: “Prioritize [action name]. This template is then combined with other elements: the farmer’s original problem and multi-source knowledge. The multi-source knowledge includes pest and disease categories with confidence levels, parameter information, and control knowledge retrieved from the knowledge graph. Finally, the combined input is used to invoke a language model, which generates treatment recommendations.

To provide a unified formulation, the recommendation process is defined as a conditional function, as shown in Equation 2:

(2) $$y_{t}=G\left(s_{t},a_{t},p_{t},k_{t},q_{t}\right)$$

where $s_{t}\in S$ denotes the predicted disease state, $a_{t}\in A_{\text{valid}}\left(s_{t}\right)$ is the selected action strategy, $p_{t}\in P$ represents contextual parameters including severity, growth stage, location, season, weather conditions, and budget, *k_t_*denotes knowledge retrieved from the knowledge graph, and *q_t_*is the farmer’s query. Here, *G*(·) is a conditional generation model. The action *a_t_*guides the generation process toward a specific intervention strategy, enabling a mapping from control strategies to treatment recommendations. The confidence score of the predicted disease is incorporated as auxiliary information to enhance reliability, but is not included in the state or parameter representation.

The agent provides treatment recommendations to farmers, who act as evaluators of the generated plans Knox and Stone (2009). Each recommendation is assessed using a multi-criteria evaluation framework Roijers et al. (2013), consisting of four criteria: treatment efficacy (*E*), economic cost (*C*), operational feasibility (*O*), and ecological safety (*S*). Each criterion is rated on a 1–5 scale.

The multi-criteria evaluations are aggregated into a scalar reward signal for unified optimization, as defined in Equation 3.

(3) $$R_{t+1}=\sum_{i=1}^{n}\omega_{i}f_{i}=\omega_{E}E+\omega_{C}C+\omega_{O}O+\omega_{S}S$$

where $f_{i}\in \left\{E,C,O,S\right\}$. The weights *ω* = {0.35,0.25,0.20,0.20} were determined based on a preliminary survey of 50 farmers, as shown in **Supplementary Table S2**. This reward signal quantifies the fit between the current strategy and actual agricultural production needs, and also provides a basis for subsequent *Q*-value iteration.

The problem is formulated as a contextual bandit, which can be viewed as a special case of Markov decision process without state transitions. Thus, the discount factor is set to *γ* = 1. The learning rate is *α* = 0.1. The agent receives a farmer reward, denoted *R_t_*_+1_. The agent then updates the *Q*-value using the *Q*-learning formula, as shown in Equation 4:

(4) $$Q\left(s_{t},a_{t}\right)\leftarrow Q\left(s_{t},a_{t}\right)+\alpha \left[R_{t+1}-Q\left(s_{t},a_{t}\right)\right]$$

The multi-criteria scores provided by end users serve as a direct measure of the effectiveness of the selected actions under specific disease conditions. This feedback mechanism not only quantifies the alignment between the current strategy and actual agricultural production needs but also provides a basis for *Q*-value iteration through immediate reward signals Ouyang et al. (2022). The agent gradually reduces the discrepancy between predicted value and actual returns, causing the distribution of actions within the policy space to converge toward maximizing user utility.

### 2.5 Construction of the dataset

The interaction dataset is directly used in the reinforcement learning interaction process. Each case is sequentially read by the agent to generate control recommendations, collect farmers’ ratings of these recommendations, and update the *Q*-table. The final saved *Q*-table is used as the reinforcement learning condition in subsequent ablation experiments.

The interaction dataset comprises 800 disease cases, each consisting of the farmer’s original inquiry, the pest or disease category and confidence level, parameter information, and the standard treatment answer. Parameter information includes severity, geographic location, season, climate, growth stage, and budget. The cases are sourced from three channels: farmer consultation records collected by agricultural extension departments over the past two years account for 60% of all cases, with all records having undergone de-identification; cases written by three plant protection experts based on simulations of all 88 disease states and their typical occurrence scenarios account for 30%; and questions crawled from public agricultural Q&A platforms and manually cleaned account for 10%. All cases were independently reviewed by two plant protection experts to ensure logical consistency between the disease and the scenario, natural language expression, and the completeness of all parameters Northcutt et al. (2021). They were then stratified and randomly sampled by crop type, disease type, severity, budget, and geographic region to ensure coverage of all 88 disease states and valid action combinations Gebru et al. (2021).

A total of 60 farmers participated in the interaction. All farmers were recruited from local agricultural cooperatives. Each farmer had at least 3 years of farming experience. Each farmer also passed a proficiency test in disease identification. The test required the correct identification of more than 20 common diseases. The accuracy rate had to be at least 80%. The farmers also passed a consistency check against expert scores. The consistency check rule was as follows: the average absolute error between experts and farmers for 5 standard cases should not exceed 1 point Artstein and Poesio (2008). As shown in **Supplementary Table S3**, all farmers received standardized training prior to the experiment. The training covered the definitions and scoring rules for the four evaluation metrics. It also included calibration exercises using 10 typical examples. Each farmer evaluated up to 30 recommendations per day. Each farmer’s individual task lasted no more than 1.5 hours. For each case, we used the mean of the combined quality scores from two farmers as the final reward signal, denoted *R_t_*_+1_. If the difference between the two farmers’ scores exceeded 1.5 points, we included a third farmer’s score. We then took the median of the three scores as *R_t_*_+ 1_.

During the preparation of the interaction dataset, a separate test set of 500 cases was constructed for evaluating the generative model. This test set is independent from the interaction dataset but covers all disease types and associated action strategies, ensuring comprehensive coverage of possible disease-action combinations.

### 2.6 Treatment recommendation generation

The treatment generation model is responsible for converting multi-source knowledge and action selection into concrete expert recommendations Yuan et al. (2024). The agent employs a dynamic prompt engineering approach based on slot filling.

The agent uses the disease name output by the visual model as the current state *s_t_*. The reinforcement learning module selects the action strategy *a_t_*based on the current state *s_t_*. The agent integrates the action strategy, the disease or pest category, parameter information, control knowledge retrieved from the knowledge graph, and the farmer’s question. The specific prompt construction logic follows the following predefined template:

Template Example: “Role: Agricultural Expert”. Prioritize: [Strategy a_t_]. Task: Treat [Disease Name & Confidence]. Parameters: [Location/Season/Weather … ]. Reference: [KG Knowledge]. Question: [Farmer Question]. Output: Personalized Advice.

The agent uses the context information derived from integrating multi-source knowledge and the action strategy as conditional constraints, and employs the language model deployed on Ollama to perform the generation task. This method helps reduce generic or vague responses. It generates treatment plans tailored to the farmer’s context, with improved accuracy and practical applicability. This process effectively mitigates the hallucination issues that may arise when language models are applied in specialized fields.

## 3 Results

### 3.1 Evaluation metrics

Six metrics are used to evaluate the performance of the visual model:

Top-1 Accuracy: The proportion of samples in which the category with the highest predicted probability matches the ground-truth category. This metric evaluates the model’s performance in identifying a single disease. A higher value indicates better performance Russakovsky et al. (2015).

Top-5 Accuracy: The proportion of samples in which the ground-truth category appears among the top five predicted categories. This metric is suitable for scenarios with many categories or easily confused classes. A higher value indicates better performance Russakovsky et al. (2015).

Top-1 Error Rate: Defined as 1 minus the Top-1 Accuracy, reflecting the model’s misclassification rate. A lower value indicates better performance Russakovsky et al. (2015).

Precision: The proportion of correctly predicted positives among all samples predicted as positive. This reflects the model’s ability to avoid false positives. A higher value indicates better performance Powers (2010).

Recall: The proportion of actual positives correctly predicted by the model. This reflects the model’s ability to avoid false negatives. A higher value indicates better performance Powers (2010).

F1-Score: The harmonic mean of precision and recall, balancing false positives and false negatives. A higher value indicates better overall classification performance Powers (2010).

Three metrics are used to evaluate the generated treatment plans: one automated metric and two evaluated by a large language model Cai et al. (2026):

BERTScore: Measures semantic similarity between generated and reference treatment plans using context-aware embeddings from a pre-trained language model. A higher score indicates better semantic fidelity Zhang et al. (2020).

Accuracy and Practicality are evaluated using DeepSeek-V4-Flash as a frozen evaluator under a predefined and consistent scoring rubricDeepSeek AI et al. (2024a). Accuracy measures the correctness of the treatment plan, while practicality evaluates its feasibility in real-world agricultural scenarios. The evaluation prompts are provided in **Table 3**. To ensure fairness and reduce evaluation bias, identical prompts are applied across all methods. The evaluator operates under a deterministic setting (temperature set to 0), and no sampling is performed during scoring.

**Table 3:** Large language model evaluation criteria for disease and pest treatment plans.

Score range	Accuracy	Practicality
4.0–5.0	The generated treatment principles, medication directions and prevention strategies are highly consistent with standard answers, complying with agricultural scientific norms with no factual or technical errors.	Detailed and practical solutions, including key details such as specific pesticide names, precise dilution ratios and critical agricultural operation steps.
3.0–3.9	General prevention directions are correct, but lack accurate pathological basis, only providing general knowledge.	Merely generic statements, lacking professional technical depth and unable to guide farmers’ on-site operations.
2.0–2.9	Obvious flaws in treatment logic or deviation from core countermeasures of standard answers.	Scant countermeasures with no executable operations.
1.0–1.9	Scientifically invalid diagnosis and treatment plans with serious misleading content.	No prevention and control measures or operational guidance provided at all.

To facilitate intuitive comparison across different evaluation criteria, all raw scores were normalized to a percentage scale (0–100%). Specifically, BERTScore was scaled as.


$$S_{\text{norm}}=S_{\text{raw}}\times 100.$$

For the LLM-based 5-point scoring system, scores were linearly rescaled using.


$$S_{\text{norm}}=\frac{S_{raw}}{5}\times 100.$$

This normalization enables consistent interpretation across metrics with different ranges.

### 3.2 Confusion matrix

It is worth noting that the visual model serves as an upstream identification module, while the main contribution of this work lies in multi-source knowledge fusion and reinforcement learning.

**Figure 5:**
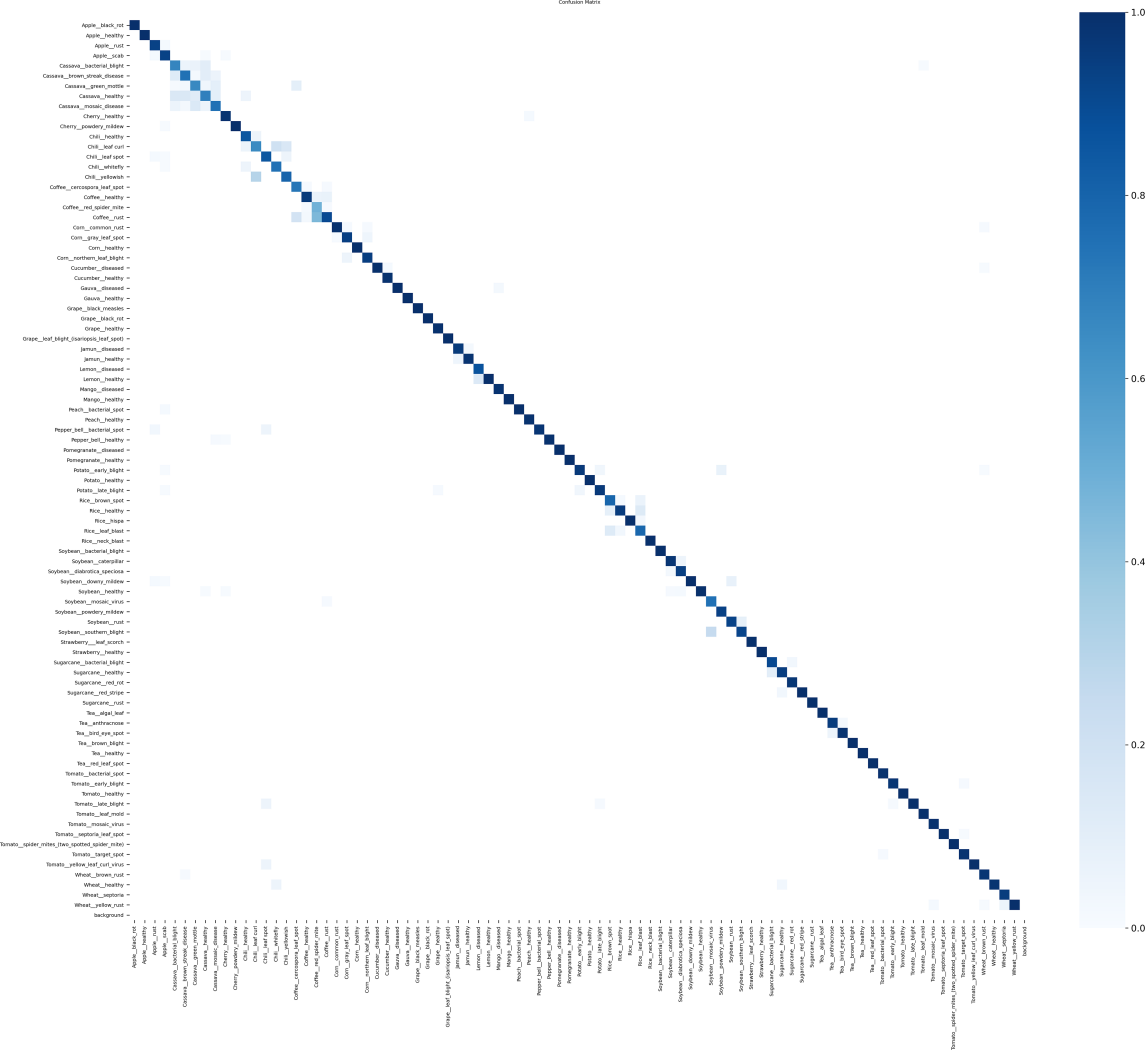
Confusion matrix for crop disease and pest identification based on the YOLO model.

The visual module employs the YOLOv8 model Upadhyay et al. (2025); Liu et al. (2024). The corresponding confusion matrix is shown in **Figure 5**. The results indicate that most categories are distributed along the diagonal, with 58 out of 88 classes achieving classification accuracy above 0.95. Only a few off-diagonal misclassifications are observed, indicating slight confusion between categories with highly similar visual features. However, these misclassifications account for only a small proportion of the total samples and have a limited impact on the overall identification performance.

### 3.3 Partial identification examples

**Figure 6:**
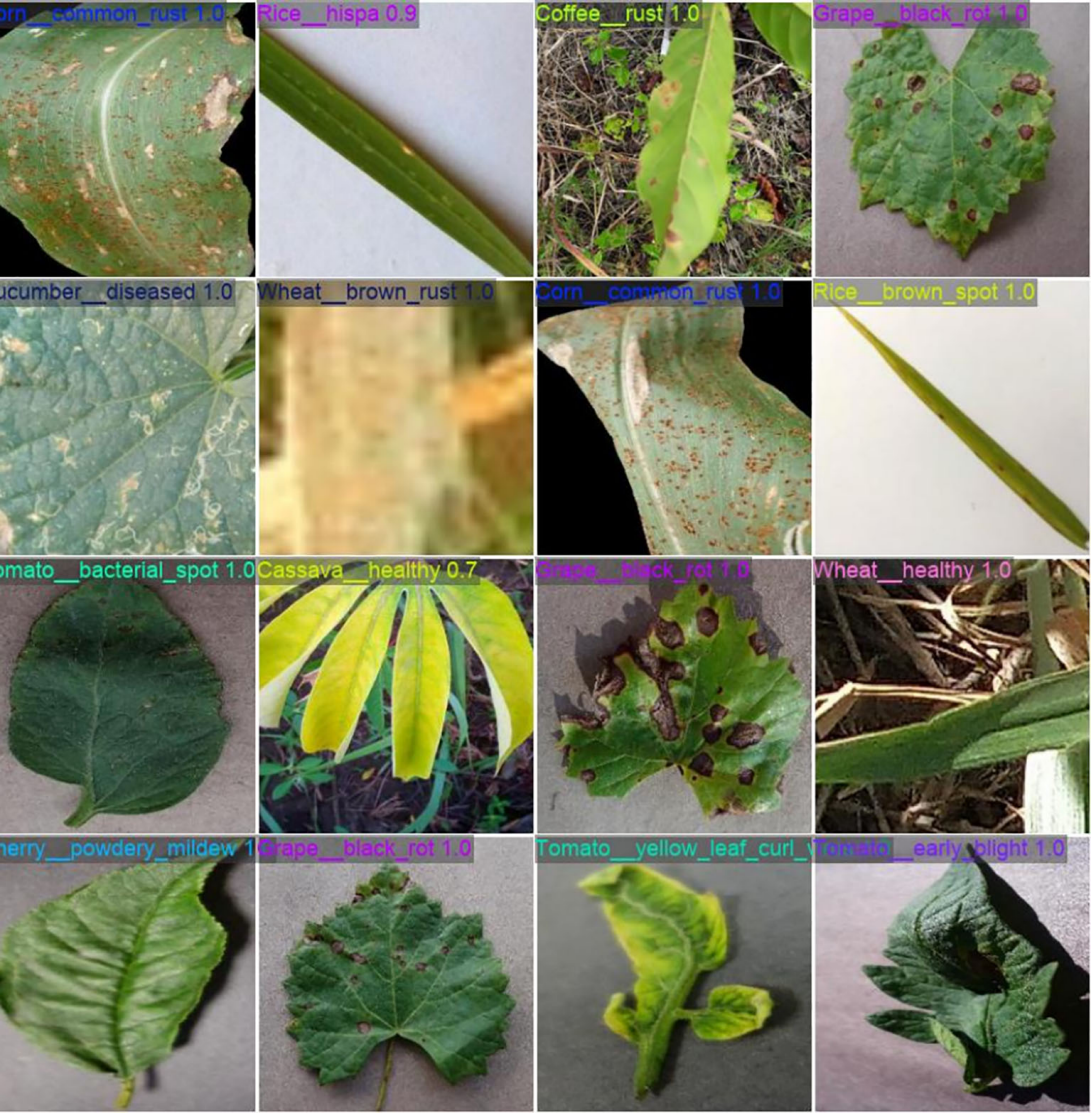
Partial results examples for crop pest/disease and healthy leaf identification using the YOLO model.

**Figure 6** presents a selection of experimental results from the YOLO-based identification task for crop pests, diseases, and healthy leaves. Each specimen is annotated with its predicted category and the corresponding confidence score. The majority of samples exhibit confidence scores between 0.9 and 1.0, with the only exception being the Cassava_healthy sample, which shows a confidence score of 0.7. These results indicate that the model consistently achieves high confidence in identifying most categories while maintaining robustness against occasional lower-confidence predictions.

### 3.4 Training, validation and test loss curves

**Figure 7:**
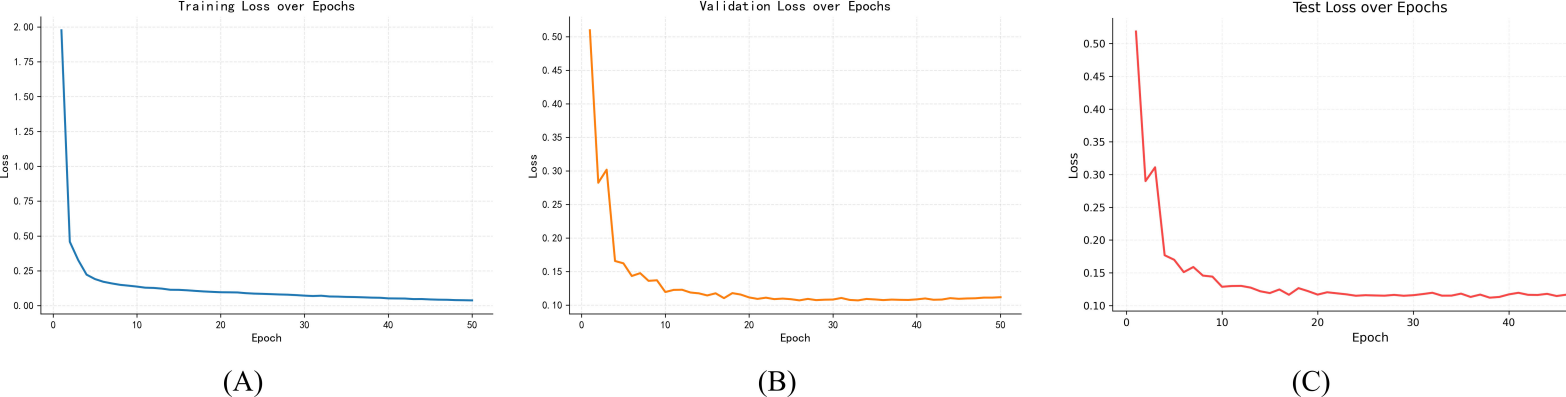
YOLO model training, validation loss and test loss curves. **(A)** Training loss, **(B)** Validation loss, **(C)** Test loss.

**Figure 7** illustrates the training, validation, and test losses across epochs. The training loss rapidly declines from approximately 2.0 to near zero within the first 30 epochs and remains approximately constant thereafter. The validation loss decreases sharply from around 0.50 to approximately 0.15 and then fluctuates slightly within a narrow range. Similarly, the test loss drops from about 0.50 to roughly 0.15 and remains stable. While the rapid convergence suggests effective learning, the near-zero training loss may indicate mild overfitting. However, the stable validation and test losses suggest that the model retains acceptable generalization performance.

### 3.5 Identification evaluation

**Figure 8:**
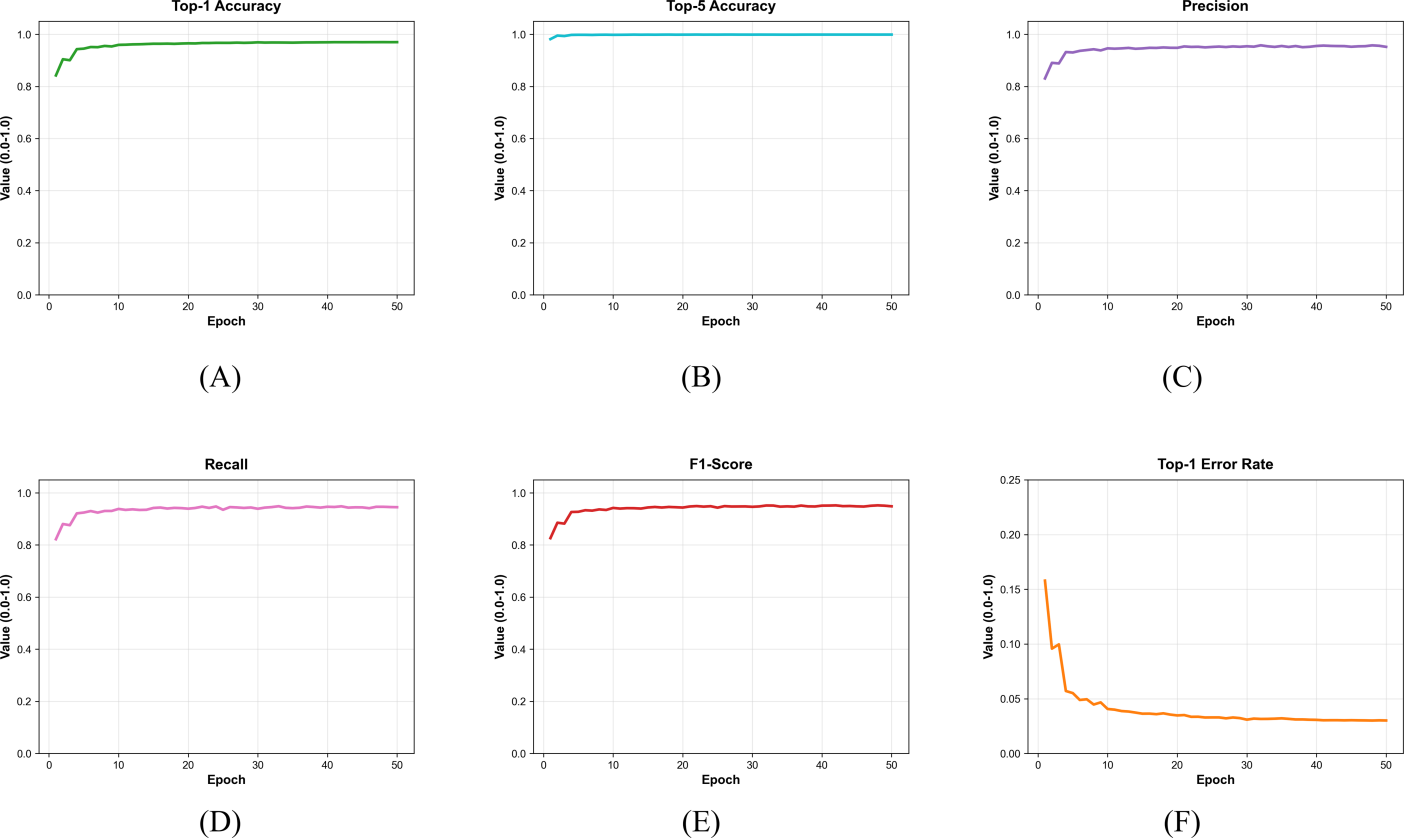
YOLO model pest and disease identification task: multiple evaluation metrics. **(A)** Top-1 Accuracy, **(B)** Top-5 Accuracy, **(C)** Precision, **(D)** Recall, **(E)** F1-Score, **(F)** Top-1 Error Rate.

**Figure 8** illustrates the evolution of six core evaluation metrics during the training of the pest and disease identification model. Top-1 Accuracy increased rapidly from approximately 0.85 and stabilized at 0.979, while Top-5 Accuracy remained largely constant at 1.000. Precision, Recall, and F1-Score converged to 0.960, 0.953, and 0.957, respectively. Overall, the model converged quickly in the early training stages, with all metrics reaching high levels and maintaining stability. These results confirm the YOLO model’s strong identification performance.

### 3.6 Q-value convergence in reinforcement learning

**Figure 9:**
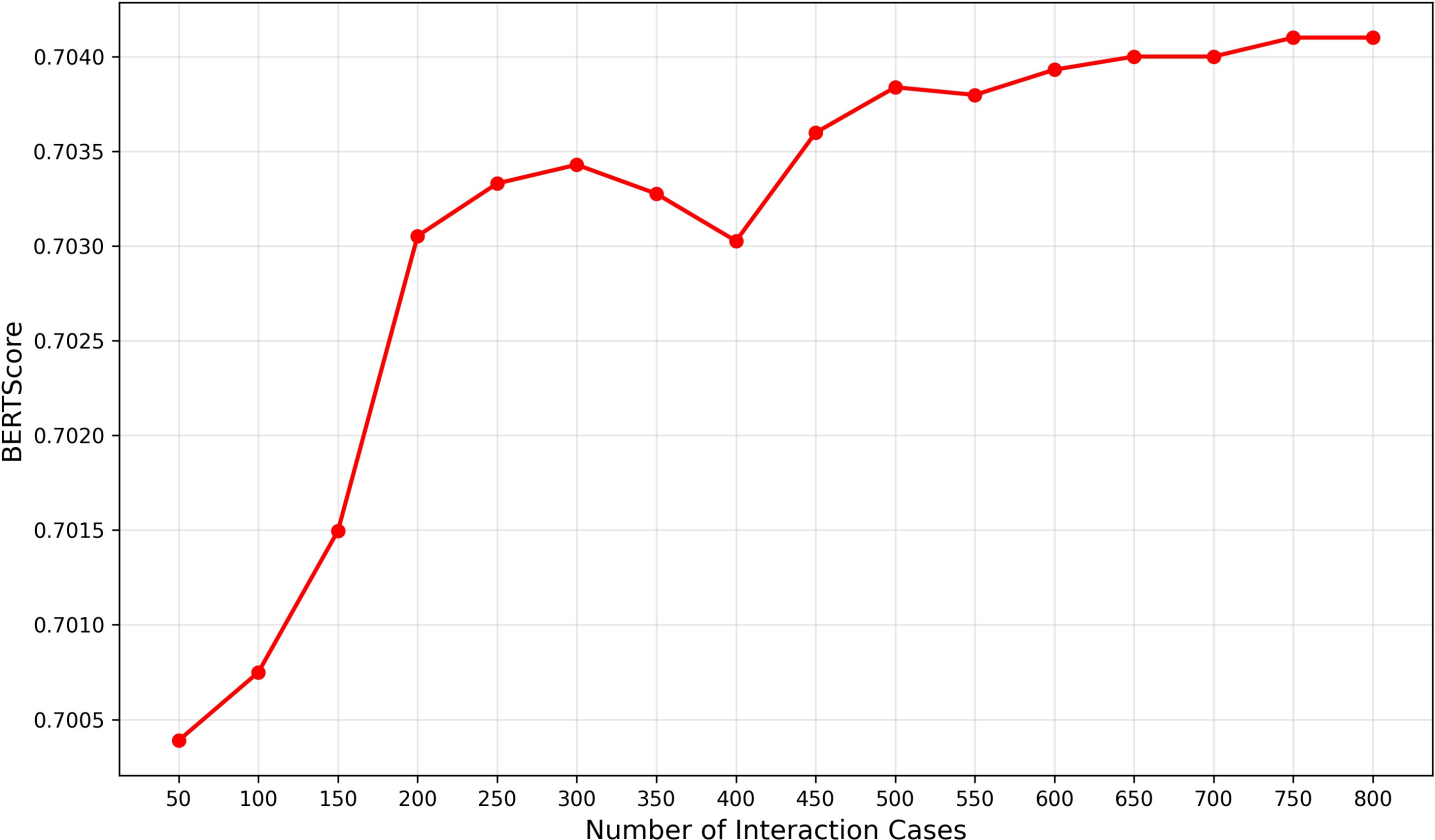
Convergence detection of the *Q*-value using a 50-case sliding-window average of BERTScore.

To monitor learning performance and convergence, a sliding-window average is employed. After processing 50 cases, the window-average BERTScore is calculated using the standard treatment answers. The *Q*-value is considered to have converged when the average BERTScore stabilizes across consecutive evaluation windows, after which the interaction is terminated. In practice, this convergence is typically reached around the 650th case; thus, using 800 cases provides a sufficient margin for convergence, as shown in **Figure 9**.

### 3.7 Multi-model evaluation of generated recommendations

Statistical significance was evaluated using one-way ANOVA followed by Tukey’s *post hoc* test, with *p<* 0.05 considered statistically significant. Results are reported as means with 95% confidence intervals.

**Figure 10:**
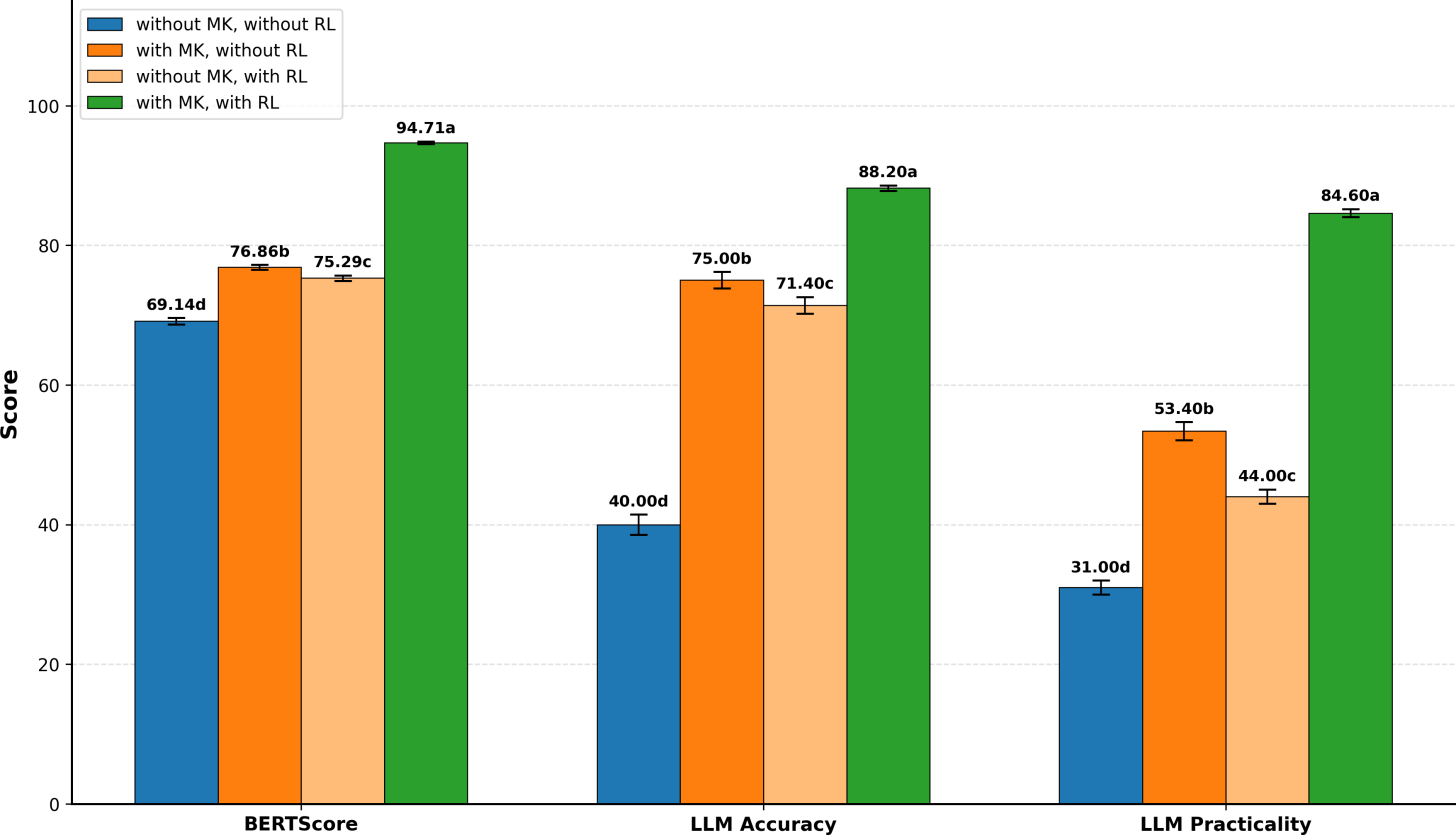
Performance comparison in Qwen-1.5B for control recommendation generation. Error bars indicate 95% confidence intervals (CI). Different lowercase letters indicate statistically significant differences among methods.

As shown in **Figure 10**, the ablation study results on the Qwen-2.5B base model demonstrate that both multi-source knowledge injection and reinforcement learning feedback can effectively improve the quality, accuracy, and practicality of the generated control plans Yang et al. (2024). Compared with the baseline, adding only multi-source knowledge or applying only reinforcement learning both yield performance improvements. The model integrating both components achieves the best performance across all three metrics: BERTScore, LLM-based accuracy and practicality. This indicates that multi-source knowledge provides the model with reliable domain background information. Specifically, contextual parameters improve situation awareness by incorporating environmental and operational constraints, while the knowledge graph enhances factual correctness by providing structured domain knowledge. Reinforcement learning, in contrast, does not introduce new knowledge but optimizes the action selection process based on farmer feedback, thereby improving the practical applicability of the generated recommendations. The generalization experiments on apple-specific diseases are presented in **Supplementary Figure S1**.

**Figure 11:**
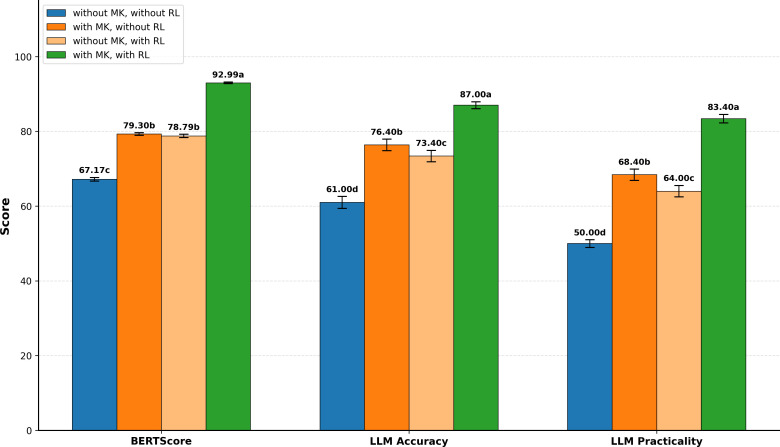
Performance comparison in Gemma-2B for control recommendation generation. Error bars indicate 95% confidence intervals (CI). Different lowercase letters indicate statistically significant differences among methods.

As shown in **Figure 11**, the Gemma-2B baseline model exhibits deficiencies in both knowledge accuracy and decision-making practicality Gemma Team (2024). After introducing multi-source knowledge and reinforcement learning, BERTScore, Accuracy, and Practicality all show significant improvements, and the generated control recommendations outperform the baseline.

**Figure 12:**
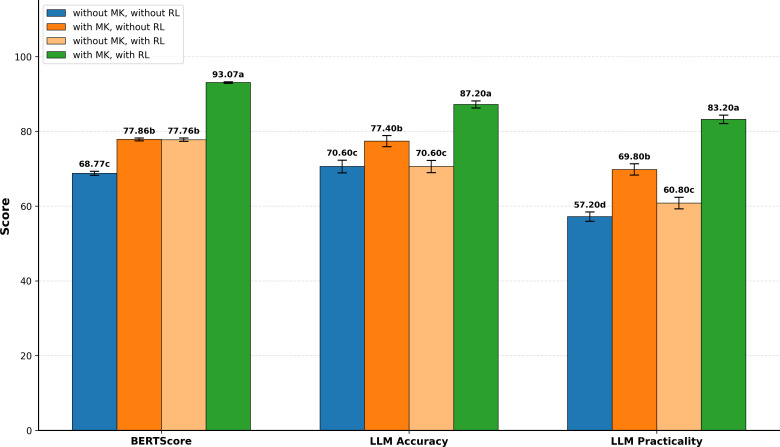
Performance comparison in LLaMA-3B for control recommendation generation. Error bars indicate 95% confidence intervals (CI). Different lowercase letters indicate statistically significant differences among methods.

As shown in **Figure 12**, the LLaMA-3B model already possesses relatively strong foundational capabilities in its baseline state Grattafiori et al. (2024). The integration of multi-source knowledge and reinforcement learning further improves performance, leading to more effective treatment recommendations.

Across the Qwen, Gemma, and LLaMA models, the independent introduction of either multi-source knowledge or reinforcement learning improves the quality of generated control recommendations. The combined use of both components consistently produces the most significant improvements, validating the effectiveness and generalization capability of the proposed approach across different base models. Importantly, the method remains highly effective even without task-specific fine-tuning data, which demonstrates strong zero-shot generalization.

## 4 Discussion

Early expert Q&A systems relied on manually compiled rules. While effective in specific scenarios, they suffered from limited knowledge coverage, high maintenance costs, and difficulties adapting to dynamic environments Sumaryanti et al. (2020). Recently, Knowledge Graphs have provided a new pathway for the systematic modeling and reasoning of agricultural knowledge. Researchers have constructed domain-specific KGs for crop management and pest diagnosis, enabling semantic interconnection and knowledge querying through entity-relationship modeling Veena et al. (2023); Zhong et al. (2025). Further studies have attempted to integrate multi-source data, such as remote sensing and soil sensors, to build spatiotemporal KGs, thereby enhancing predictive capabilities for pest occurrence patterns and propagation risks Zhang et al. (2023); Li et al. (2024). However, existing systems still face several limitations in supporting precise end-to-end decision-making. Firstly, most KGs lack the support of real-time dynamic data, leading to generation capabilities that become detached from specific environmental contexts and unable to adapt to rapid changes in field conditions Yan et al. (2025); Zheleva et al. (2017). Secondly, there is a failure to deeply fuse user subjective preferences, real-time objective parameters, and disease information from the KG. Consequently, generating highly personalized and actionable treatment recommendations remains challenging Zhai et al. (2020); Singh et al. (2023). To address these deficiencies, this paper proposes a knowledge-enhanced framework for multi-source knowledge fusion. The agent fuses high-confidence visual identification results, multi-factor contextual parameters, and diagnostic advice through KG retrieval. Ultimately, using designed, structured prompt templates, these diverse knowledge sources are integrated into a coherent context, directly driving the language model to generate scenario-specific, feasible solutions. This approach transitions from static knowledge retrieval to dynamic situational generation, effectively bridging gaps in accuracy and practicality in current systems.

In the domain of intelligent agricultural decision-making, Reinforcement Learning has been applied to optimize pest control. Research primarily focuses on learning pesticide application strategies in simulated environments Yang et al. (2025) or integrating crop growth models to plan long-term management schemes Turchetta et al. (2022). Their reward functions typically rely on predefined quantitative metrics derived from models Stetter et al. (2024); Sharifi et al. (2025). However, these static, preset reward functions fail to capture the complex, comprehensive assessments made by real farmers regarding control efficacy, economic cost, operational feasibility, and ecological safety Masere and Worth (2022); Finger et al. (2019). Furthermore, learning processes that rely entirely on simulation environments lack closed-loop feedback from real farmers and field systems, leading to insufficient practical applicability and adaptability of the strategies Shadkam and Irannezhad (2025). Simultaneously, the decision-making process lacks interpretability, which may reduce farmer trust and adoption Chen et al. (2022); Angelov et al. (2021). To address these shortcomings, this paper proposes a reinforcement learning approach based on farmer evaluation feedback. By directly converting farmers’ actual evaluations of control efficacy, economic cost, operational feasibility, and ecological safety into dynamic reward signals, the agent continuously optimizes itself through interaction with the real environment, ensuring that optimization objectives align with genuine farmer needs.

## 5 Conclusion

This study demonstrates that an intelligent agricultural decision-making agent integrating multi-source knowledge and reinforcement learning can improve decision quality in dynamic agricultural environments. Multi-source knowledge fusion improves the accuracy of treatment recommendations in complex scenarios, while reinforcement learning guided by multi-criteria farmer feedback enables continuous adaptation under real-world production constraints, resulting in recommendations that are more consistent with practical agricultural needs.

Despite these advantages, the current study still faces limitations in generalization, particularly under rare sample distributions and extreme weather conditions. Future work will focus on improving cross-regional generalization by incorporating multi-region datasets, including agricultural data from regions such as India and Brazil. In addition, SHAP-based explainable methods will be introduced to enhance interpretability, transparency, and the robustness of reinforcement learning-based decision-making across diverse agricultural environments.

## Data Availability

The raw data supporting the conclusions of this article will be made available by the authors, without undue reservation.

## References

[B1] Aijaz N. Lan H. Raza T. Yaqub M. Iqbal R. Pathan M. S. (2025). Artificial intelligence in agriculture: Advancing crop productivity and sustainability. J. Agric. Food Res. 20, 101762. doi: 10.1016/j.jafr.2025.101762 38826717

[B2] Albahli S. (2025). Agrifusionnet: A lightweight deep learning model for multisource plant disease diagnosis. Agriculture 15, 1523. doi: 10.3390/agriculture15141523 30654563

[B3] Amaral G. Rodrigues O. Simperl E. (2024). Prove: A pipeline for automated provenance verification of knowledge graphs against textual sources. Semant. Web 15, 2159–2192. doi: 10.3233/SW-233467

[B4] Angelov P. P. Soares E. A. Jiang R. Arnold N. I. Atkinson P. M. (2021). Explainable artificial intelligence: an analytical review. Wiley. Interdiscip. Rev.: Data Min. Knowled. Discov. 11, e1424. doi: 10.1002/widm.1424 41531421

[B5] Araujo S. O. Peres R. S. Ramalho J. C. Lidon F. Barata J. (2023). Machine learning applications in agriculture: current trends, challenges, and future perspectives. Agronomy 13, 2976. doi: 10.3390/agronomy13122976 30654563

[B7] Artstein R. Poesio M. (2008). Inter-coder agreement for computational linguistics. Comput. Linguist. 34, 555–596. doi: 10.1162/coli.07-034-R2

[B8] Ban T. Wang X. Chen L. Wu X. Chen Q. Chen H. (2022). Quality evaluation of triples in knowledge graph by incorporating internal with external consistency. IEEE Trans. Neural Networks Learn. Syst. 35, 1980–1992. doi: 10.1109/TNNLS.2022.3186033 35786563

[B9] Breure T. S. Estrada-Carmona N. Petsakos A. Gotor E. Jansen B. Groot J. C. (2024). A systematic review of the methodology of trade-off analysis in agriculture. Nat. Food 5, 211–220. doi: 10.1038/s43016-024-00926-x 38443487 PMC10963264

[B10] Cai G. Tian R. Yang L. Jia Y. Li L. Wang J. (2026). Efficient inference for edge large language models: A survey. Tsinghua. Sci. Technol. 31, 1365–1380. doi: 10.26599/TST.2025.9010166

[B11] Carvalho F. P. (2017). Pesticides, environment, and food safety. Food Energy Secur. 6, 48–60. doi: 10.1002/fes3.108 41531421

[B12] Chen V. Li J. Kim J. S. Plumb G. Talwalkar A. (2022). Interpretable machine learning: Moving from mythos to diagnostics. Commun. ACM 65, 43–50. doi: 10.1145/3546036

[B13] DeepSeek AI Liu A. Feng B. Xue B. Wang B. Wu B. . (2024). DeepSeek-V3 technical report (Ithaca, NY, USA: DeepSeek AI). doi: 10.48550/arXiv.2412.19437

[B14] Dobrovsky A. (2021). Plant disease classification merged dataset. Dataset deposited in Kaggle repository, version 1.0. (San Francisco, CA, USA: Kaggle (Google LLC)).

[B15] Eastwood C. Klerkx L. Ayre M. Dela Rue B. (2019). Managing socio-ethical challenges in the development of smart farming: from a fragmented to a comprehensive approach for responsible research and innovation. J. Agric. Environ. Ethics. 32, 741–768. doi: 10.1007/s10806-017-9704-5 30311153

[B16] Fayaz S. Jahangeer Sidiq S. Zaman M. Butt M. (2022). Machine learning: An introduction to reinforcement learning. Mach. Learn. Data Sci.: Fundament. Appl., 1–22. doi: 10.1002/9781119776499.ch1 41531421

[B17] Finger R. Swinton S. M. El Benni N. Walter A. (2019). Precision farming at the nexus of agricultural production and the environment. Annu. Rev. Res. Econ 11, 313–335. doi: 10.1146/annurev-resource-100518-093929 41139587

[B18] Gautron R. Baudry D. Adam M. Falconnier G. N. Hoogenboom G. King B. . (2024). A new adaptive identification strategy of best crop management with farmers. Field Crops Res. 307, 109249. doi: 10.1016/j.fcr.2024.109249 38826717

[B19] Gebru T. Morgenstern J. Vecchione B. Vaughan J. W. Wallach H. Daumé H. III . (2021). Datasheets for datasets. Commun. ACM 64, 86–92. doi: 10.1145/3458723

[B20] Gemma Team (2024). Gemma: open models based on gemini research and technology (Mountain View, CA, USA: Google DeepMind). doi: 10.48550/arXiv.2403.08295

[B21] Godfray H. C. J. Beddington J. R. Crute I. R. Haddad L. Lawrence D. Muir J. F. . (2010). Food security: the challenge of feeding 9 billion people. Science 327, 812–818. doi: 10.1126/science.1185383 20110467

[B31] Grattafiori A. Dubey A. Jauhri A. Pandey A. Kadian A. Al-Dahle A. . (2024). The llama 3 herd of models (Ithaca, NY, USA: Meta). doi: 10.48550/arXiv.2407.21783

[B22] Iaksch J. Fernandes E. Borsato M. (2021). Digitalization and big data in smart farming–a review. J. Manage. Analyt. 8, 333–349. doi: 10.1080/23270012.2021.1897957 37339054

[B23] Kamilaris A. Prenafeta-Boldú F. X. (2018). Deep learning in agriculture: A survey. Comput. Electron. Agric. 147, 70–90. doi: 10.1016/j.compag.2018.02.016 38826717

[B24] Klerkx L. Jakku E. Labarthe P. (2019). A review of social science on digital agriculture, smart farming and agriculture 4.0: New contributions and a future research agenda. NJAS. Wageningen. J. Life Sci. 90, 100315. doi: 10.1016/j.njas.2019.100315 38826717

[B25] Knox W. B. Stone P. (2009). “ Interactively shaping agents via human reinforcement: The tamer framework”, in: Proceedings of the Fifth International Conference on Knowledge Capture (K-CAP '09), (New York, NY, USA: ACM), 9–16. doi: 10.1145/1597735.1597738

[B26] Li H. Tan B. Sun L. Liu H. Zhang H. Liu B. (2024). Multi-source image fusion based regional classification method for apple diseases and pests. Appl. Sci. 14, 7695. doi: 10.3390/app14177695 30654563

[B27] Liakos K. G. Busato P. Moshou D. Pearson S. Bochtis D. (2018). Machine learning in agriculture: A review. Sensors 18, 8. doi: 10.3390/s18082674 30110960 PMC6111295

[B28] Liu J. Wang X. (2024). Multisource information fusion method for vegetable disease detection. BMC Plant Biol. 24, 738. doi: 10.1186/s12870-024-05346-4 39095689 PMC11295898

[B29] Liu Y. Yu Q. Geng S. (2024). Real-time and lightweight detection of grape diseases based on fusion transformer yolo. Front. Plant Sci. 15, 1269423. doi: 10.3389/fpls.2024.1269423 38463562 PMC10920279

[B30] Masere T. P. Worth S. H. (2022). Factors influencing adoption, innovation of new technology and decision-making by small-scale resource constrained farmers: The perspective of farmers in lower gweru, Zimbabwe. Afr. J. Food Agric. Nutr. Dev. 22, 20013–20035. doi: 10.18697/ajfand.108.20960

[B32] Mohanty S. P. Hughes D. P. Salath M. (2016). Using deep learning for image-based plant disease detection. Front. Plant Sci. 7, 215232. doi: 10.3389/fpls.2016.01419 27713752 PMC5032846

[B33] Northcutt C. G. Jiang L. Chuang I. L. (2021). Confident learning: Estimating uncertainty in dataset labels. J. Artif. Intell. Res. 70, 1373–1411. doi: 10.1613/jair.1.12125

[B34] Ouyang L. Wu J. Jiang X. Almeida D. Wainwright C. Mishkin P. . (2022). “ Training language models to follow instructions with human feedback”, in: Advances in Neural Information Processing Systems. arXiv preprint (2203.02155), (Ithaca, NY, USA: Curran Associates, Inc), 27730–27744. doi: 10.48550/arXiv.2203.02155

[B50] Peng L. Yang P. Ye J. Li Y. (2026). The construction and refined extraction techniques of knowledge graph based on large language models. Sci. Rep. 16, 8104. doi: 10.1038/s41598-026-38066-w 41667618 PMC12960662

[B35] Powers D. M. (2010). Evaluation: from precision, recall and f-measure to roc, informedness, markedness and correlation. arXiv. doi: 10.48550/arXiv.2010.16061

[B36] Ray D. K. Mueller N. D. West P. C. Foley J. A. (2013). Yield trends are insufficient to double global crop production by 2050. PloS One 8, e66428. doi: 10.1371/journal.pone.0066428 23840465 PMC3686737

[B37] Roijers D. M. Vamplew P. Whiteson S. Dazeley R. (2013). A survey of multi-objective sequential decision-making. J. Artif. Intell. Res. 48, 67–113. doi: 10.1613/jair.3987

[B38] Russakovsky O. Deng J. Su H. Krause J. Satheesh S. Ma S. . (2015). Imagenet large scale visual recognition challenge. Int. J. Comput. Vision 115, 211–252. doi: 10.1007/s11263-015-0816-y 30311153

[B6] Saccone D. Vallino E. (2025). Global food security in a turbulent world: reviewing the impacts of the pandemic, the war and climate change. Agric. Food Econ. 13, 47. doi: 10.1186/s40100-025-00388-0

[B39] Savary S. Willocquet L. Pethybridge S. J. Esker P. McRoberts N. Nelson A. (2019). The global burden of pathogens and pests on major food crops. Nat. Ecol. Evol. 3, 430–439. doi: 10.1038/s41559-018-0793-y 30718852

[B40] Shadkam E. Irannezhad E. (2025). A comprehensive review of simulation optimization methods in agricultural supply chains and transition towards an agent-based intelligent digital framework for agriculture 4.0. Eng. Appl. Artif. Intell. 143, 109930. doi: 10.1016/j.engappai.2024.109930 38826717

[B41] Sharifi A. Migliorini S. Quaglia D. (2025). Optimizing trajectories for rechargeable agricultural robots in greenhouse climatic sensing using deep reinforcement learning with proximal policy optimization algorithm. Future Internet 17, 1–21. doi: 10.3390/fi17070296 30654563

[B42] Singh N. Sunitha N. Tripathi G. Saikanth D. Sharma A. Jose A. E. . (2023). Impact of digital technologies in agricultural extension. Asian J. Agric. Extens. Econ Sociol. 41, 963–970. doi: 10.9734/ajaees/2023/v41i92127 42216225

[B43] Stetter C. Huber R. Finger R. (2024). Agricultural land use modeling and climate change adaptation: A reinforcement learning approach. Appl. Econ. Perspect. Policy 46, 2040–5790. doi: 10.1002/aepp.13448 41531421

[B44] Sumaryanti L. Istanto T. Pare S. (2020). “ Rule based method in expert system for detection pests and diseases of corn”, in: Journal of Physics: Conference Series (Bristol, UK: IOP Publishing), 1569.

[B45] Taghizadeh-Hesary F. Rasoulinezhad E. Yoshino N. (2019). Energy and food security: Linkages through price volatility. Energy Policy 128, 796–806. doi: 10.1016/j.enpol.2018.12.043 38826717

[B46] Tang F. H. Lenzen M. McBratney A. Maggi F. (2021). Risk of pesticide pollution at the global scale. Nat. Geosci. 14, 206–210. doi: 10.1038/s41561-021-00712-5 37880705

[B47] Turchetta M. Corinzia L. Sussex S. Burton A. Herrera J. Athanasiadis I. . (2022). “ Learning long-term crop management strategies with cyclesgym”, in: Advances in Neural Information Processing Systems (La Jolla, CA, USA: Curran Associates, Inc) 35, 11396–11409.

[B48] Upadhyay A. Chandel N. S. Singh K. P. Chakraborty S. K. Nandede B. M. Kumar M. . (2025). Deep learning and computer vision in plant disease detection: a comprehensive review of techniques, models, and trends in precision agriculture. Artif. Intell. Rev. 58, 92. doi: 10.1007/s10462-024-11100-x 30311153

[B49] Veena G. Gupta D. Kanjirangat V. (2023). Semi-supervised bootstrapped syntax-semantics-based approach for agriculture relation extraction for knowledge graph creation and reasoning. IEEE Access 11, 138375–138398. doi: 10.1109/ACCESS.2023.3339552 25079929

[B51] Yan R. An P. Meng X. Li Y. Li D. Xu F. . (2025). A knowledge graph for crop diseases and pests in China. Sci. Data 12, 222. doi: 10.1038/s41597-025-04492-0 39915513 PMC11802884

[B53] Yang R. Li B. Dong J. Cai Z. Lin H. Wang F. . (2025). Reinforcement learning-based generative artificial intelligence for novel pesticide design. J. Adv. Res. 78, 179–190. doi: 10.1016/j.jare.2025.02.030 40032026 PMC12685504

[B52] Yang A. Yang B. Zhang B. Hui B. Zheng B. Yu B. . (2024). Qwen2.5 technical report. arXiv. doi: 10.48550/arXiv.2412.15115

[B54] Yuan Z. Liu K. Peng R. Li S. Leybourne D. Musa N. . (2024). Pestgpt: Leveraging large language models and iot for timely and customized recommendation generation in sustainable pest management. IEEE Internet Things. Mag. 8, 26–33. doi: 10.1109/IOTM.001.2400036 25079929

[B55] Zhai Z. Martínez J. F. Beltran V. Martínez N. L. (2020). Decision support systems for agriculture 4.0: Survey and challenges. Comput. Electron. Agric. 170, 105256. doi: 10.1016/j.compag.2020.105256 38826717

[B59] Zhang X. Chen Y. Li Z. (2024). A novel method for boosting knowledge representation learning in entity alignment through triple confidence. Mathematics 12, 1214. doi: 10.3390/math12081214 30654563

[B57] Zhang T. Kishore V. Wu F. Weinberger K. Q. Artzi Y. (2020). “ Bertscore: Evaluating text generation with bert”, in: International Conference on Learning Representations (La Jolla, CA, USA: Curran Associates, Inc.) 2020, 568–579. doi: 10.48550/arXiv.1904.09675

[B58] Zhang W. Peng L. Ge X. Yang L. Chen L. Li W. (2023). Spatio-temporal knowledge graph-based research on agro-meteorological disaster monitoring. Remote Sens. 15, 4403. doi: 10.3390/rs15184403 30654563

[B56] Zhang G. Xiong Y.-J. Hu J.-P. Xia C.-M. (2025). Triplet trustworthiness validation with knowledge graph reasoning. Eng. Appl. Artif. Intell. 141, 109813. doi: 10.1016/j.engappai.2024.109813 38826717

[B61] Zhao X. Chen B. Ji M. Wang X. Yan Y. Zhang J. . (2024). Implementation of large language models and agricultural knowledge graphs for efficient plant disease detection. Agriculture 14, 1359. doi: 10.3390/agriculture14081359 30654563

[B60] Zhao C. Liu B. Piao S. Wang X. Lobell D. B. Huang Y. . (2017). Temperature increase reduces global yields of major crops in four independent estimates. Proc. Natl. Acad. Sci. 114, 9326–9331. doi: 10.1073/pnas.1701762114 28811375 PMC5584412

[B62] Zheleva M. Bogdanov P. Zois D.-S. Xiong W. Chandra R. Kimball M. (2017). “ Smallholder agriculture in the information age: Limits and opportunities”, in: Proceedings of the 2017 Workshop on Computing within Limits (New York, NY, USA: ACM), 59–70.

[B63] Zhong M. Wei L. Mo H. (2025). Cotton pest and disease diagnosis via yolov11-based deep learning and knowledge graphs: a real-time voice-enabled edge solution. Front. Plant Sci. 16, 1671755. doi: 10.3389/fpls.2025.1671755 41140380 PMC12546045

