# Supplementary material for: A multi-source fusion and feedback-optimized intelligent agent for crop disease and pest diagnosis and treatment
Source: Front Plant Sci. 2026 Jun 19;17:1811772. doi: 10.3389/fpls.2026.1811772 (PMC13328098; doi:10.3389/fpls.2026.1811772)
Supplement: Supplementary file 1 [file DataSheet1.pdf]

Supplementary Material

1 SUPPLEMENTARY TABLES AND FIGURES

The knowledge graph data for this study were sourced from authoritative channels, including the Government Service Platform of the Ministry of Agriculture and Rural Affairs and the National Agricultural Technology Extension Service Center. The data cover the general framework for pest and disease control, basic information on crop diseases, occurrence regions and periods, prevention and control plans, pesticide application specifications, and knowledge related to special economic crops. These multi-source data provide a reliable basis for extracting entities, relationships, and attributes in the knowledge graph, as shown in Table S1.

The weights of the four evaluation indicators for farmer feedback in this study were determined through a preliminary questionnaire survey involving 50 frontline farmers. Detailed statistical results are provided in Table S2. A total of 50 questionnaires were distributed, and all were returned. All respondents were frontline farmers with at least 1 year of experience in field planting and pest or disease control, ensuring the sample's representativeness. A 1-5 point scale was used to rate the importance of each indicator. After calculating the average scores, normalized weights were derived to better reflect actual agricultural production needs, thereby providing a reliable basis for constructing the reward function. Additionally, Table S3 details the scoring rules used by farmers to evaluate generated treatment plans.

As shown in Fig. S1, to further evaluate the generalization of the proposed method, we conducted experiments on three Apple-specific diseases: Black Rot, Scab, and Rust, using the Qwen-2.5B base model. The results show that both multi-source knowledge and reinforcement learning independently improve BERTScore, LLM-based accuracy, and practicality, and their combination achieves the highest performance across all three diseases. These findings indicate that the proposed method maintains robust generalization even on individual crop categories, supporting its effectiveness in zero-shot scenarios without task-specific training data.

Table S1. Sources of the Knowledge Graph for Crop Pest and Disease Control

Knowledge Category	Source	Data Application Content
General framework for pest and disease control	Regulations on the Prevention and Control of Crop Diseases and Pests	Provides the official national framework and general principles for crop pest and disease control.
Basic information on pests and diseases	Government Service Platform of the Ministry of Agriculture and Rural Affairs; Tutorial on Standard Cultivation Techni ques for Fruit Trees and Cash Crops	Extracts common pest and disease types, occurrence patterns, identification features, occurrence conditions, and basic control points for fruit trees and food crops.
Occurrence regions and periods of pests and diseases	National Agricultural Technology Extension Service Center	Extracts major occurrence regions, peak periods, and key environmental conditions for epidemics of various pests and diseases.
Prevention and control plans for pests and diseases	Department of Crop Production, Ministry of Agriculture and Rural Affairs; Official Website of the National Agricultural Technology Extension Service Center	Extracts control plans for major crop pests and diseases and agricultural pests, and supplements official recommended control strategies for different crops.
Pesticide application specifications	Guidelines for the Rational Use of Pesticides	Specifies applicable diseases, application concentrations, application methods, and safety precautions for commonly used pesticides.
Knowledge related to special economic crops	National Agricultural Technology Extension Service Center	Supplements suitable planting regions, sowing periods, common pests and diseases, and supporting control plans for special economic crops.

Table S2. Farmer Questionnaire Survey on the Weights of Four Evaluation Indicators

Evaluation Dimension	Symbol	Average Farmer Score (1–5 scale)	Normalized Weight
Treatment efficacy	E	4.38	0.35
Economic cost	C	3.75	0.25
Operational feasibility	O	3.00	0.20
Ecological safety	S	3.00	0.20

Table S3. Farmer Scoring Rules for the generated treatment plans

Dimension	Score 1	Score 2	Score 3	Score 4	Score 5
Treatment efficacy (E)	Completely ineffective	Very poor effect	Moderate effect	Good effect	Excellent effect
Economic cost (C)	Extremely high cost	Relatively high cost	Moderate cost	Low cost	Very low cost
Operational feasibility (O)	Extremely complex	Difficult to operate	Operable but with ambiguous steps	Specific procedures	Very simple
Ecological safety (S)	Severe harm to the environment	Obvious adverse effects	Moderate impact	Minor impact	Completely harmless

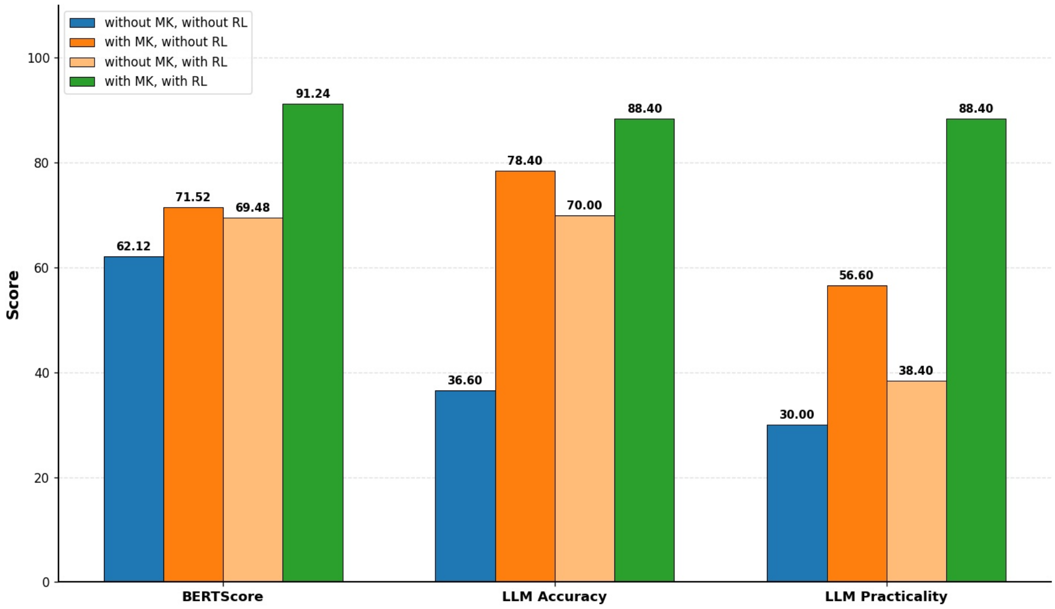

Figure S1: Performance of Qwen-2.5B on Apple-specific diseases (Black Rot, Scab, and Rust) under different configurations. Metrics include BERTScore, LLM-based Accuracy, and Practicality. MK = multi-source knowledge, RL = reinforcement learning.
